# A network perspective on the evolution of metabolism by gene duplication

**DOI:** 10.1186/gb-2007-8-2-r26

**Published:** 2007-02-27

**Authors:** Juan Javier Díaz-Mejía, Ernesto Pérez-Rueda, Lorenzo Segovia

**Affiliations:** 1 Departamento de Ingeniería Celular y Biocatálisis, Instituto de Biotecnología, Universidad Nacional Autónoma de México. Av. Universidad 2001, Col. Chamilpa, Cuernavaca, Morelos, CP 62210 México

## Abstract

**Background:**

Gene duplication followed by divergence is one of the main sources of metabolic versatility. The patchwork and stepwise models of metabolic evolution help us to understand these processes, but their assumptions are relatively simplistic. We used a network-based approach to determine the influence of metabolic constraints on the retention of duplicated genes.

**Results:**

We detected duplicated genes by looking for enzymes sharing homologous domains and uncovered an increased retention of duplicates for enzymes catalyzing consecutive reactions, as illustrated by the ligases acting in the biosynthesis of peptidoglycan. As a consequence, metabolic networks show a high retention of duplicates within functional modules, and we found a preferential biochemical coupling of reactions that partially explains this bias. A similar situation was found in enzyme-enzyme interaction networks, but not in interaction networks of non-enzymatic proteins or gene transcriptional regulatory networks, suggesting that the retention of duplicates results from the biochemical rules governing substrate-enzyme-product relationships. We confirmed a high retention of duplicates between chemically similar reactions, as illustrated by fatty-acid metabolism. The retention of duplicates between chemically dissimilar reactions is, however, also greater than expected by chance. Finally, we detected a significant retention of duplicates as groups, instead of single pairs.

**Conclusion:**

Our results indicate that *in silico *modeling of the origin and evolution of metabolism is improved by the inclusion of specific functional constraints, such as the preferential biochemical coupling of reactions. We suggest that the stepwise and patchwork models are not independent of each other: in fact, the network perspective enables us to reconcile and combine these models.

## Abstract 2

*In silico *models trying to explain the origin and evolution of metabolism are improved with the inclusion of specific functional constraints, such as the preferential coupling of reactions.

## Background

The classical view of metabolism is that relatively isolated sets of reactions or pathways allow the synthesis and degradation of compounds. The new perspective views metabolic components (substrates, products, cofactors, and enzymes) as parts of a single network. Defining metabolism as pathways is not always straightforward because some functional properties, such as the smaller distances between reactions from different pathways are visible only when metabolism is analyzed from a network perspective [1]. A way to do this is to represent metabolism with a compound-centric network, wherein nodes (substrates and products) participating in the same reaction are connected. Alternatively, in an enzyme-centric network, nodes (enzymes) producing a compound are connected with nodes consuming the same compound. These tools have shown that metabolism has a scale-free topology [2,3], meaning that the majority of nodes show a low degree of connectivity and the topology of the network is dominated by a small fraction of highly connected nodes. Another property of metabolic networks is their hierarchical modularity [4,5], showing groups of highly clustered, functionally related nodes.

**Figure 1:**
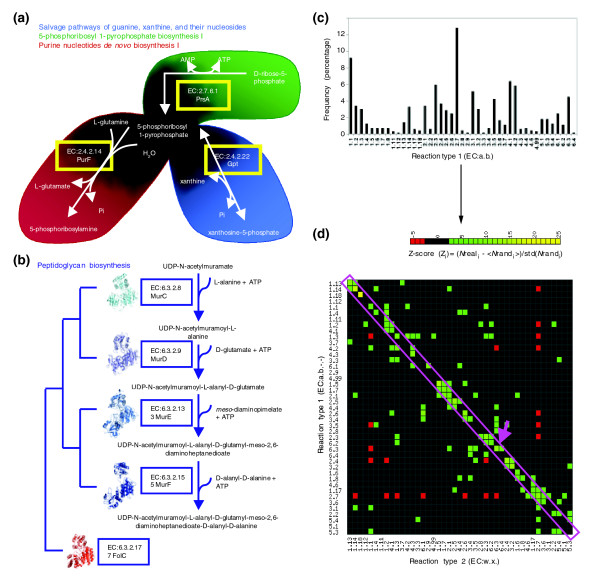
Preferential biochemical coupling of reactions in metabolic networks. **(a) **Homologous transferases PurF and Gpt from *E. coli *catalyze consecutive chemically similar reactions. Their origin can be explained by both the stepwise and the patchwork models. **(b) **Homologous ligases involved in peptidoglycan biosynthesis whose origin can be explained by both the stepwise and the patchwork models. A distant homolog (FolC) acts in folate metabolism. **(c) **Frequencies of reaction types (EC:a.b) in the *E. coli *K12 metabolic network, according to KEGG (hereafter called EcoKegg). **(d) **Frequencies of consecutive reaction types (EC:a.b → EC:w.x) in EcoKegg were compared against the expected values using a set of null Maslov-Sneppen models (see Materials and methods). The Z-score (color-scale bar at top) indicates the number of standard deviations between the real and the average expected frequencies. Consecutive reaction types overrepresented in real networks are shown in green-to-yellow, underrepresented ones are shown in red. The diagonal (pink box) highlights consecutive chemically similar reactions, including the ligases synthesizing peptidoglycan (pink arrow). Reaction types were sorted vertically using a hierarchical clustering to detect highly related reaction types, such as EC:1.5, EC:1.7 and EC:2.1. (center of plot).

Recent models have successfully simulated the origin of scale-free networks by gene duplication [6], while their modular organization has been explained by the preferential attachment of new nodes to the most highly connected preexisting ones [5]. These models do not, however, take into account the functional constraints of metabolism [6]. For instance, carbon-nitrogen ligases (EC:6.3) tend to act consecutively, reducing their chance of associating with enzymes catalyzing other reaction types (Figure 1). We call this property 'preferential biochemical coupling of reactions', and suggest that it reflects a biochemical necessity - in the synthesis of the peptidoglycan of bacterial cell walls, for example. Our results show the importance of including functional constraints to improve models of the origin and evolution of metabolic networks. Indeed, a recent model simulating the origin of highly connected compounds in metabolic networks [7] is significantly improved when reactions are considered as coupled pairs instead of single entities.

The first hypotheses on the origin and evolution of enzyme-driven metabolism were based on the idea that gene duplication, followed by divergence, can lead to the origin of new metabolic reactions. The two pioneering models - 'stepwise' [8] (or retrograde) and 'patchwork' [3] evolution - have two main differences. The stepwise model posits that, in the case where a substrate tends to be depleted, gene duplication can provide an enzyme capable of supplying the exhausted substrate, giving rise to homologous enzymes catalyzing consecutive reactions. The patchwork model, on the other hand, postulates that duplication of genes encoding promiscuous enzymes (capable of catalyzing various reactions) allows each descendant enzyme to specialize in one of the ancestral reactions. In this regard, enzymes generated by patchwork evolution can catalyze reactions a greater distance apart in the pathway than those originated by stepwise evolution. The second difference is that the stepwise model invokes consecutive reactions and so can originate enzymes catalyzing chemically dissimilar reactions (CDRs) but preserving specificity for the type of substrate [9,10]. In contrast, the patchwork model considers that promiscuous enzymes tend to catalyze chemically similar reactions (CSRs) even while acting on different types of substrates [9,10]. A simple way to find whether enzymes catalyze similar reactions is to compare the first two digits of their EC numbers (EC:a.b) [10-12].

Some authors have used the differences between the stepwise and patchwork models in an attempt to clarify their contributions to specific instances of evolution of metabolism. Collectively, these analyses suggest the patchwork model as the most common mechanism generating metabolic versatility [9-12]. A major difficulty with these analyses is the significant fraction of consecutive and chemically similar reactions that are catalyzed by homologous enzymes [10,11]. Because they are consecutive, the stepwise model could explain the origin of such reactions, but the patchwork model can also explain them because they are chemically similar. For example, amidophosphoribosyl transferase and xanthine phosphoribosyltransferase are homologous enzymes catalyzing consecutive reactions and so their origin could be attributed to the stepwise model. They catalyze CSRs, however, and so their origin could also be explained by the patchwork model (Figure 1a). Similarly, the origin of four homologous carbon-nitrogen ligases catalyzing consecutive reactions in peptidoglycan biosynthesis is consistent with both the stepwise and patchwork models [10] (Figure 1b). In the work reported here we have determined that the fraction of consecutive CSRs in metabolism is significantly greater than expected by chance, implying that the origin of such reactions can be explained by the complementary actions of stepwise and patchwork evolution. We suggest that a network-based approach can reconcile these two models.

In this article we reconstruct the enzyme-centric metabolic networks of *Escherichia coli *K12 and a number of other organisms using information from the BioCyc [13,14] and KEGG [15] databases. The protein sequences of the enzymes were compared to detect duplicated genes, which we shall call 'duplicates'. We evaluated the influence of both chemical similarity and the distance between reactions (for example, the number of reactions that separate them) on the rate of retention of duplicates. We also estimated whether the preferential biochemical coupling of reactions and the modularity of networks affect this rate. Finally, we detected cases in which duplicates have been retained as groups and determined how general this is.

## Results and discussion

### The preferential biochemical coupling of reactions in metabolic networks reflects a functional constraint

Metabolism follows logical rules that imply that specific reactions and fluxes are temporally and spatially compartmentalized [16]. We searched for some of these rules in our data, determining whether the combination of reaction types (each designated as EC:a.b) is constrained by biochemical necessity or is simply the result of random processes. To do this, we determined the frequency of paired reaction types for a large set of different metabolic networks and compared it against the value expected by chance. To calculate these expected values a set of null Maslov-Sneppen models [17] was generated. The models are randomly rewired versions of the original network, preserving the degree of connectivity for each node (see Materials and methods). The results show that certain reaction types tend to occur consecutively (Figure 1d). As an illustration of the biological relevance of this finding, consider the case of carbon-nitrogen ligases (EC:6.3), which tend to be followed by other EC:6.3 enzymes, for example in the synthesis of peptidoglycan (Figure 1b). In fact, a recent study uncovers that metabolites also show a preferential coupling [18]. We consider that these biases reflect underlying biochemical mechanisms and the need for particular substrate stoichiometries. In the following sections we discuss the relevance of this finding to the retention of duplicates.

### Influence of chemical similarity on the retention of duplicates

**Figure 2:**
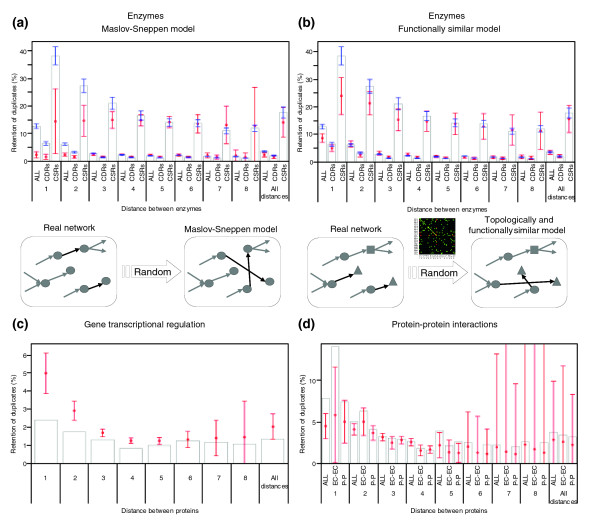
Influence of chemical similarity and distance on the retention of duplicates. **(a) **Frequencies of retained duplicates (histogram bars) in EcoKegg are shown for the whole reaction set (ALL), and the subsets of chemically similar reactions (CSRs) and chemically different reactions (CDRs) at different distances (metabolic steps). Blue bars indicate three standard deviations (σ) from these frequencies. Deviations were obtained by random sampling. Red dots represent the average expected frequencies ± 3σ obtained using Maslov-Sneppen models. The rewiring to construct the null model is shown below the graph. **(b) **A similar procedure to (a) was carried out, using null functionally similar models to control the influence of the preferential biochemical coupling of reactions. Symbols as in (a). Compared with Maslov-Sneppen models, in which all nodes are equally eligible for change, in functionally similar models the preferential biochemical coupling of reactions restricts the choices. **(c) **Retention of duplicates in the gene regulatory network of *E. coli *as a function of the distance (number of regulatory interactions) between transcription factors and target genes. **(d) **Retention of duplicates in a protein-protein interaction network of *E. coli*. The full set of interactions (ALL), and the subsets of enzyme-enzyme (EC-EC) and non-enzymatic protein-protein (P-P) interactions are shown. In (c) and (d) red dots represent averages obtained using Maslov-Sneppen models.

We computed the frequency of retention of duplicates for both CSRs and CDRs. The frequencies were then compared against the values expected by chance, using Maslov-Sneppen models, to determine whether they can be attributed to biological pressure. Figure 2a shows that retention of duplicates between CSRs is sixfold greater than between CDRs. This agrees with previous reports [10-12]. Note, however, that for both CSRs and CDRs, duplicates separated by less than three nodes in a network are more frequent than expected by chance (Z-score > 3, *P *< 0.001). The main implication of this finding is that for both CSRs and CDRs the retention of duplicates is not random, but reflects underlying biological phenomena. Thus, gene duplication is an important source of metabolic variability and also of biochemical innovations.

### Influence of distance between reactions on the retention of duplicates

In addition to the retention of duplicates generating CSRs and CDRs, Figure 2a shows an increased retention of duplicates between reactions at smaller distances apart. The explanation of this phenomenon is non-trivial because there is no biological trait clearly associable to a shorter distance between reactions. We therefore compared the results from metabolic networks with those from other biological networks to determine whether our observation is general. We identified duplicates within a gene regulatory network [19] and within a validated protein-protein interaction network [20], both from *E. coli*. The regulatory network did not show a significant influence of the distance between transcription factors and target genes on the retention of duplicates (Figure 2c). In contrast, the protein-protein interaction network (Figure 2d) shows an increased retention of duplicates between proteins at smaller distances from each other in the network. A more detailed analysis shows that this increase is mainly due to enzyme-enzyme interactions. In fact, the fraction of non-enzymatic duplicates, mainly comprising protein complexes involved in DNA replication, transcription, translation, and protein folding, is not significantly different from random (Z-score < 3, *P *> 0.001). Thus, it seems that the increased retention of duplicates between proteins at smaller distances apart in the network is characteristic of metabolic networks and enzyme-enzyme complexes. From this observation, we propose that laws governing substrate-enzyme-product relationships in metabolic networks are different from those acting on protein-DNA and non-enzymatic protein-protein interactions. A possible reason for this is that in metabolic interactions proteins interact with small molecules as substrates and products, whereas non-enzymatic protein-protein and protein-DNA interactions require larger interacting protein surfaces, and their retention could be more difficult. In fact, some authors have shown that regulatory protein-DNA interactions are quickly lost [21]. In contrast, protein-protein interactions are preserved in a higher degree, in particular those involved in metabolic processes [22].

What are the factors distinguishing metabolic networks from other types of biological networks that could increase the retention of duplicates between nodes at smaller distances apart to each other? We found that the preferential biochemical coupling of reactions is an important constraint characteristic of metabolic networks and so we simulated the retention of duplicates in a set of 'functionally' similar null models including this constraint. These models are rewired versions of the original network, preserving both the degree of connectivity and the preferential biochemical coupling of reactions, as described in Materials and methods. The retention of duplicates simulated using Maslov-Sneppen models (red circles in Figure 2a) shows a behavior independent of the distance between proteins. In contrast, using the functionally similar models (red circles in Figure 2b) an increased retention of duplicates between nodes at smaller distances apart was detected, better approximating what happens in real metabolic networks. This implies that the preferential biochemical coupling of reactions partially explains the increased retention of duplicates between reactions at smaller distances apart to each other. Because this coupling of reactions is exclusive to metabolism, this finding also helps us to understand why this behavior was not detected in transcriptional regulatory and non-enzymatic protein-protein interaction networks.

Finally, we controlled for various network and enzyme properties on the retention of duplicates. First, we considered whether the increased retention of duplicates is restricted to a region of the network. To evaluate this we randomly sampled the network and computed the retention of duplicates within samples. The main finding (blue bars in Figure 1a,b) is that the increased retention of duplicates between reactions at smaller distances apart to each other remains statistically significant (Z-score > 3, *P *< 0.001), and is not restricted to a region of the network. Second, we evaluated the influence of highly promiscuous compounds (hubs) on the retention of duplicates, gradually excluding hubs from network reconstructions and computing the retention of duplicates each time. The increased retention of duplicates between enzymes at smaller distances apart in the network remains statistically significant (Z-score > 3, *P *< 0.001) (see Additional data file 4). Similar results were found on analyzing different metabolic networks (see Additional data file 4). Third, because a significant number of enzymes consist of two or more domains, having only one EC number assigned, and vice versa [23], their direct comparison can cause false positives. To avoid this, we manually split enzyme sequences by functional domains. In addition, in one control (see Additional data file 5), we extracted the subset of single-domain enzymes and repeated the analyses of retention of duplicates. In a second control (see Additional data file 5), we required that all domains between duplicates are homologous. The results from these two controls support the ones discussed above. Fourth, we redefined our criterion of chemical similarity, using both the first digit of EC numbers (EC:a) and the first three digits (EC:a.b.c). As expected, these new criteria modify the relative rates of retained duplicates in CSRs and CDRs (see Additional data file 5), but the increased retention of duplicates at smaller distances apart to each other remains significant, supporting our previous conclusions. Finally, because we used a method to detect remote homology (based on hidden Markov models), we controlled for this method conducting a search for homologs using BLAST (which detects more closely related homologs) and PSI-BLAST (remotely related homologs) (Additional data file 5). As expected, the rate of retained duplicates changes when considering only closely related homologous, but the increased retention of duplicates between reactions at smaller distances apart remains statistically significant (Z-score > 3, *P *< 0.001). Collectively, these controls indicate that the increased retention of duplicates at smaller distances apart is independent of the way in which metabolic databases are constructed, their size, and the hub prevalence. The manual validation of enzyme domains and network databases could give our findings more precision, but the main conclusions are robust.

### Influence of network modularity on retention of duplicates

**Figure 3:**
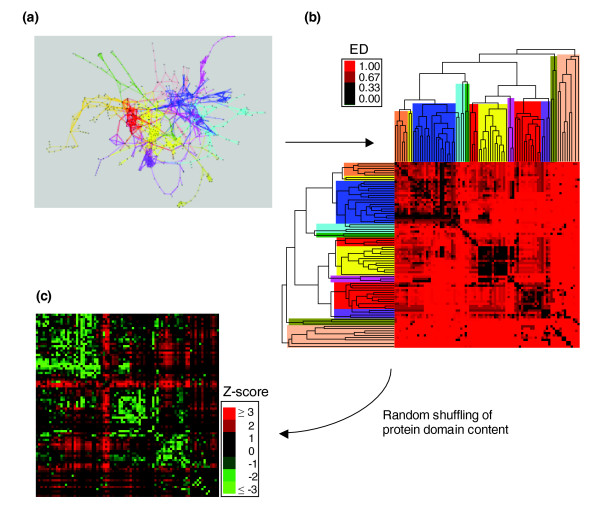
Influence of network modularity on the retention of duplicates. **(a) **A hierarchical clustering was carried out to delimit modules in metabolic networks. Colors denote different modules in EcoKegg. **(b) **Metabolic pathways (branches in the trees) within and across modules were compared using a measure of evolutionary distance (ED). Modules comprising related branches are indicated by color as in (a). A value of (ED) closer to zero (the darker squares) implies a greater retention of duplicates between the two given pathways. **(c) **Observed (ED) values were compared against those expected by chance - after random shuffling of protein-domains. A Z-score < -3 (green) refers to significant (ED) values (*P *< 0.001).

Metabolic networks have been reported to possess modular architecture [4,5]. Enzymes constituting a module are highly clustered neighbors, and consequently one could expect a higher retention of duplicates within modules than between them. To test this hypothesis we used a hierarchical clustering algorithm to detect modules in metabolic networks (Figure 3a, and see Materials and methods). Then we calculated a paired measure of evolutionary distance (ED) for all-against-all metabolic pathways. This measure reflects the retention of duplicates between pathways within and between modules. Our definition of (ED) is similar to the one used to determine the relatedness between genomes based on protein-domain content [24] (see Materials and methods). Note that (ED) is not the distance referred to in previous sections, which was the distance between nodes in the network. The results show that metabolic pathways of the same module tend to have a lower (ED) (Figure 3b). This implies a greater retention of duplicates within modules than between them. For instance, considering the *E. coli *metabolic network as a whole, the total retention of duplicates among CSRs is around 15%. In contrast, if one module is extracted, such as amino-acid metabolism (colored blue in Figure 3a,b), and the retention of duplicates within it is calculated, the resulting fraction is around 50%. To assess the significance of (ED) values we compared them against those expected by chance. To do this, we simulated a null scenario preserving both the connectivity and interaction partners of the original network, but the domain content across proteins was randomly shuffled (see Materials and methods). This analysis shows that the retention of duplicates within modules is significantly greater than between them (Z-score > 3, *P *< 0.001) (Figure 3c). Thus, we propose that the capability of metabolic networks to grow modularly by gene duplication is highly related to two factors: the closeness together of reactions; and the kind of substrate(s) participating within each module. Further studies evaluating the influence of metabolite similarity on the retention of duplicates could help to understand this phenomenon.

### Retention of duplicates as groups and single entities

**Figure 4:**
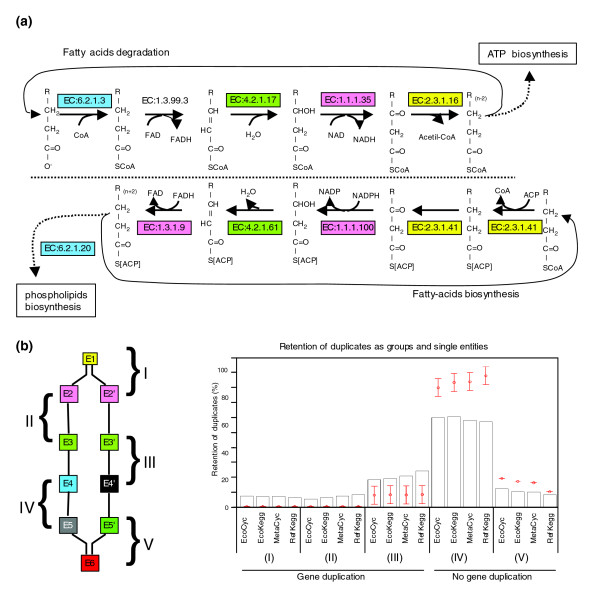
Retention of duplicates as groups and single entities. **(a) **The fatty-acid degradative and biosynthetic routes illustrate the retention of duplicates as groups. The same colors in EC number boxes denote duplicates. **(b) **Retention of duplicates acting consecutively. Five hypothetical scenarios were analyzed (left panel). Boxes of the same color denote duplicates. The number and letter (for example, E2 and E2') indicate the place of the reaction in the series. Scenarios (I) and (V) have a common reaction followed or preceded by two possible reactions. In (I) gene duplication was detected, in (V) it was not. Scenarios (II), (III) and (IV) involve pairs of consecutive reactions in two branches of the network. In (II) both pairs are duplicates, in (III) only one pair is duplicated, and in (IV) none of the pairs are duplicates. From this diagram one can see that one pair can participate in more than one scenario, looking upstream or downstream in the network flux. The histogram on the right shows the frequency for each scenario. We present the results for the four databases analyzed herein. The networks were reconstructed eliminating the top 20 hubs. These results are the comparison of all-against-all pairs (EC:a.b → EC:w.x), including CSRs as well as CDRs. Red dots represent the expected average frequencies ± 3σ obtained using Maslov-Sneppen models.

Finally, we determined the frequency of duplicates retained as groups (pairs of consecutive reactions), instead of single entities. To illustrate this idea, consider fatty-acid degradation (β-oxidation) and biosynthesis (Figure 4a). These pathways are chemically similar, but act in opposite directions and differ in their acyl-carrier groups. We determined that enzymes catalyzing CSRs in these pathways originated by gene duplication. Thus, we suggest that an ancestral pathway catalyzed both fatty-acid degradation and biosynthesis. The direction of this ancestral pathway would be dependent on the acyl carriers and fatty acids available. To get a first approximation of the generality of this observation, we carried out an all-against-all comparison of the enzymes catalyzing consecutive CSRs (EC:a.b → EC:w.x). Our results indicate that about 15% of enzymes have at least one homolog in a metabolic pathway. Of these, two thirds are retained as isolated duplicates (scenario III in Figure 4b) and a third are retained as groups (scenario II in Figure 4b). Interestingly, the retention of both groups and isolated duplicates is greater than expected by chance (Z-scores > 50). In contrast, non-retention of duplicates is lower than expected (Z-score < -20). We therefore suggest that models trying to explain the increase in the complexity of metabolism by gene duplication should include the retention of both groups and isolated duplicates.

## Conclusion

We used an enzyme-centric network approach to estimate the retention of duplicates in metabolism using information from various sources (multiple species and various databases). The observed frequencies were compared against null models to determine their significance. Collectively, our results highlight the influence of both distance apart in the network and chemical similarity of reactions on the retention of duplicates. Specifically, we found an increased retention of duplicates between consecutive reactions (Figure 2a,b), and show that this bias can be partially attributed to the preferential biochemical coupling of reactions (Figure 2b). A similar analysis using gene regulatory and protein-protein interaction networks shows that this behavior is characteristic of enzymatic relationships. Thus, we propose that the laws governing substrate-enzyme-product interactions are different from those acting on protein-DNA and non-enzymatic protein-protein interactions (Figure 2c,d). This is reflected as a higher retention of duplicates within a network module than between modules (Figure 3). In addition, our results show a significant retention of duplicates acting on both CSRs and CDRs (Figure 2), supporting the idea that gene duplication is important in generating innovations as well as metabolic variants [9-12]. A synergy between closeness in the network and chemical similarity between reactions explains the high retention of duplicates between consecutive CSRs (Figure 2a). Our hypothesis that duplicates are significantly retained as groups can be extended to several series of reactions (Figure 4).

We therefore consider that gene duplication should be studied as a single process, instead of distinguishing separate stepwise and patchwork models. The difficulties that arise from traditional conceptions of these models are avoided with the network-based approach used here, which reconciles the two.

Biological networks share general topological properties, such as their scale-free behavior and hierarchical modularity. In fact, some of these properties have been found in social and technological networks [2,5,19,25,26]. Our findings agree with previous studies suggesting that the next step in modeling the origin and evolution of networks must consider not only the properties they share but also those that differentiate them [7,25,27]. In particular, we have improved the modeling of metabolic networks by including the preferential biochemical coupling of reactions. A more detailed analysis looking at other functional constraints, such as metabolite similarity and binding versus catalytic enzyme properties, as well as massive gene duplications and horizontal gene transfer, could enhance our understanding of the influence of metabolic versatility in the evolution of species.

## Materials and methods

### Network reconstruction

Enzyme-centric metabolic networks were reconstructed according to two databases BioCyc v8.0 (EcoCyc and MetaCyc) and KEGG v0.4 (EcoKegg and the full KEGG, refered RefKegg) as follow. If reaction R1 produces the compound A, and A is the substrate of R2, a directed link between the EC numbers of R1 and R2 was established. In reversible reactions, a second link, from the EC number of R2 to the EC number of R1, was added. To obtain information about reactions from BioCyc the following files were used: reactions.dat (substrate/product), enzrxns.dat (reversibility) and reaction-links.dat (EC numbers). The xml files from KEGG provide similar information in their sections reaction (substrate/product and reversibility) and entries id (EC numbers). Hubs were detected for each network, and the links established solely by hubs were gradually eliminated. The reconstructed networks, eliminating the top 20 hubs, possess the following number of nodes and edges: EcoCyc (976/4,473), EcoKegg (804/2,410), MetaCyc (964/4,230), RefKegg (2575/11,499).

### Detection of retained duplicates

Enzyme sequences were retrieved, according to the desired EC number, from the following databases: EcoCyc, UNIPROT [28], BRENDA [29], and KEGG. A manual split of sequences by functional domains, according to UNIPROT, was carried out to avoid false positives caused by multifunctional enzyme comparisons. The final set has 4,534 domain sequences, representing 1,527 EC numbers completely annotated and 348 partial annotations. To detect duplicates, sequences were compared against the hidden Markov models of homolog domains of SUPERFAMILY v1.65 [30] and PFAM v16 [31] databases. The HMMER v2.3.1 suite of programs [32] was used for this comparison, with an E-value = 0.001 as threshold. We assumed as chemically similar those reactions catalyzed by enzymes whose EC numbers share the first two digits (EC:a.b). A network adjacency matrix containing every pair of nodes (i,j) was subjected to the Floyd-Warshall algorithm [33] to determine the distance (minimal path length) between each pair (i,j). The adjacency matrix contained all reactions with known substrate/products, including those without an assigned enzyme (gene). This strategy permits us to determine the retention of duplicates as a function of both the distance apart in the network and the chemical similarity between reactions. The function (1/distance_ij_^2^) was used to construct a matrix of normalized associations for all pairs (*i*,*j*). This matrix was used to perform a hierarchical clustering to detect network modules. To do this, we used the Kendall's τ algorithm implemented in the program CLUSTER 3.0 [34]. Similar results were obtained using the Spearman rank correlation. To determine the retention of duplicates within and between modules we calculated the evolutionary distance (ED) for each pair of pathways as follows:

(ED) = A'/(A' + AB)

where A' is the number of enzymes of the smaller pathway (pA) without homologs in the second pathway (pB). AB is the number of enzymes of pA with homologs in pB. At one extreme, when all the enzymes of pA have homologs in pB, the evolutionary distance converges on 0. In contrast, when the two pathways share no homologs the value of evolutionary distance converges on 1.

### Significance tests

To determine whether the higher retention of duplicates between reactions at smaller distances apart could be restricted to a portion of the network we conducted 10,000 half-random samplings of the real network and calculated the frequency of retained duplicates within each sample. In addition, we determined the significance of these frequencies, comparing them against the values expected by chance using two sets of null models. The first, comprising 10,000 Maslov-Sneppen models, preserve the degree of connectivity for each node of the original network, but edges were randomly rewired. To construct these models, two edges of the original network were randomly chosen and their inputs were switched. This was repeated until the original network was completely rewired (see lower panel of Figure 2a). The second set, comprising 10,000 'functionally' similar models, preserves both the degree of connectivity and the preferential biochemical coupling of reactions of the original network. To construct these models, two edges of the original network were randomly chosen, but their inputs were switched only if both the inputting and outputting nodes represent chemically similar reactions (see lower panel of Figure 2b). Otherwise, another two edges were chosen, and the former ones were returned for further choices. This was repeated until the network was completely rewired. Some edges, from chemically similar groups with an even number of pairs, remain unpaired after rewiring their group. They were added to models in their original form. These pairs represent less than 5% of the models.

We used the Z-score (Z_i_) to determine the significance of real frequencies as follows:

*Z*_*i *_= (*N*real_i _- <*N*rand_i_>)/std(*N*rand_i_)

where *N*real_i _is the frequency of an attribute (i) in the real network. For example, the frequency for each reaction-type pair, the number of retained duplicates at a given distance, and so on. <*N*rand_i_> and std(*N*rand_i_) are the average frequency and standard deviation of (i) in null models. A Z-score ≥ 3 implies that the frequency of (i) in the real network is significantly greater than expected by chance (*P *< 0.001). In contrast a Z-score ≤ -3 indicates that (i) is significantly underrepresented in the real network.

To determine the significance of evolutionary distances within and between modules, we compared the actual values against the ones expected using 1,000 null models. These models preserve the networks intact (connectivity and wiring), but the domain content was shuffled across proteins. A Z-score ≤ -3 implies that retention of duplicates between two pathways is greater than expected by chance (*P *< 0.001).

## Additional data files

The following additional data are available online with this paper. Additional data file 1 shows the reconstructed metabolic networks from various databases (EcoKegg, EcoCyc, RefKegg and MetaCyc), eliminating hubs gradually in each database. Additional data file 2 shows the amino-acid sequences of the enzymes analyzed in this work. Additional data file 3 shows the domains detected in such sequences, grouped by EC numbers. Additional data file 4 shows the results of retention of duplicates in various databases, gradually removing hubs. Additional data file 5 shows the controls for the multidomain enzymes, the criteria of chemical similarity, and the method used to detect duplicates.

## Supplementary Material

Additional data file 1 Reconstructed metabolic networks from various databases (EcoKegg, EcoCyc, RefKegg and MetaCyc), eliminating hubs gradually in each database. Click here for file

Additional data file 2 Amino-acid sequences of the enzymes analyzed in this work. Click here for file

Additional data file 3 Domains detected in the amino-acid sequences of the enzymes analyzed, grouped by EC numbers. Click here for file

Additional data file 4 Results of retention of duplicates in various databases, gradually removing hubs. Click here for file

Additional data file 5 Controls for the multidomain enzymes, the criteria of chemical similarity, and the method used to detect duplicates. Click here for file
