# Supplementary material for: A network perspective on the evolution of metabolism by gene duplication
Source: Genome Biol. 2007 Feb 27;8(2):R26. doi: 10.1186/gb-2007-8-2-r26 (PMC1852415; doi:10.1186/gb-2007-8-2-r26)
Supplement: Additional data file 4 — Results of retention of duplicates in various databases, gradually removing hubs. [file gb-2007-8-2-r26-S4.ppt]

## Slide 1
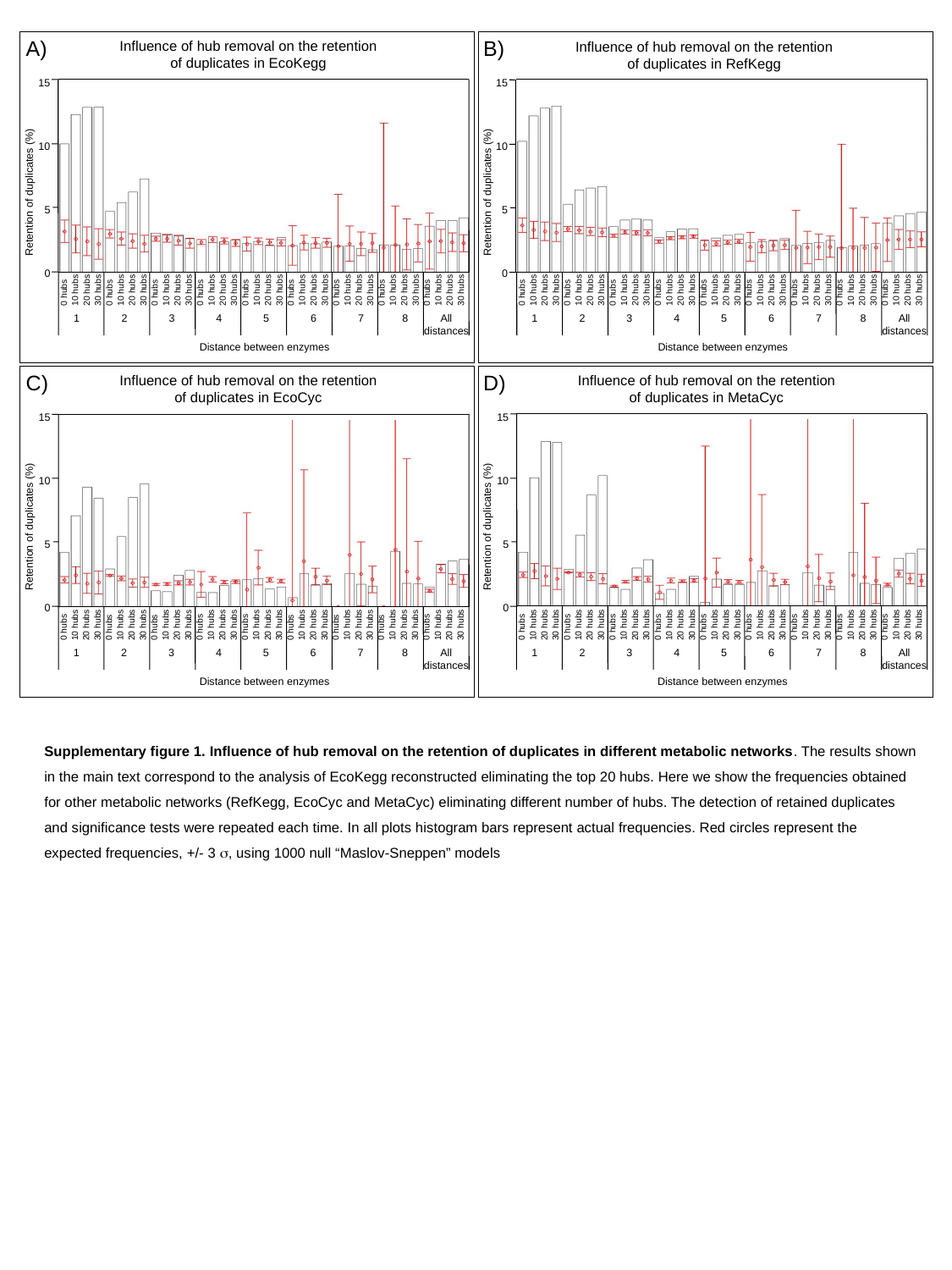

A)
B)
Influence of hub removal on the retention
of duplicates in EcoKegg
15
10
5
0
0 hubs
10 hubs
20 hubs
30 hubs
0 hubs
10 hubs
20 hubs
30 hubs
0 hubs
10 hubs
20 hubs
30 hubs
0 hubs
10 hubs
20 hubs
30 hubs
0 hubs
10 hubs
20 hubs
30 hubs
0 hubs
10 hubs
20 hubs
30 hubs
0 hubs
10 hubs
20 hubs
30 hubs
0 hubs
10 hubs
20 hubs
30 hubs
0 hubs
10 hubs
20 hubs
30 hubs
Retention of duplicates (%)
2 3 4 5 6 7 8
All
distances
Distance between enzymes
Influence of hub removal on the retention
of duplicates in RefKegg
15
10
5
0
0 hubs
10 hubs
20 hubs
30 hubs
0 hubs
10 hubs
20 hubs
30 hubs
0 hubs
10 hubs
20 hubs
30 hubs
0 hubs
10 hubs
20 hubs
30 hubs
0 hubs
10 hubs
20 hubs
30 hubs
0 hubs
10 hubs
20 hubs
30 hubs
0 hubs
10 hubs
20 hubs
30 hubs
0 hubs
10 hubs
20 hubs
30 hubs
0 hubs
10 hubs
20 hubs
30 hubs
Retention of duplicates (%)
2 3 4 5 6 7 8
All
distances
Distance between enzymes
C)
D)
Influence of hub removal on the retention
of duplicates in MetaCyc
15
10
5
0
0 hubs
10 hubs
20 hubs
30 hubs
0 hubs
10 hubs
20 hubs
30 hubs
0 hubs
10 hubs
20 hubs
30 hubs
0 hubs
10 hubs
20 hubs
30 hubs
0 hubs
10 hubs
20 hubs
30 hubs
0 hubs
10 hubs
20 hubs
30 hubs
0 hubs
10 hubs
20 hubs
30 hubs
0 hubs
10 hubs
20 hubs
30 hubs
0 hubs
10 hubs
20 hubs
30 hubs
Retention of duplicates (%)
2 3 4 5 6 7 8
All
distances
Distance between enzymes
Influence of hub removal on the retention
of duplicates in EcoCyc
15
10
5
0
0 hubs
10 hubs
20 hubs
30 hubs
0 hubs
10 hubs
20 hubs
30 hubs
0 hubs
10 hubs
20 hubs
30 hubs
0 hubs
10 hubs
20 hubs
30 hubs
0 hubs
10 hubs
20 hubs
30 hubs
0 hubs
10 hubs
20 hubs
30 hubs
0 hubs
10 hubs
20 hubs
30 hubs
0 hubs
10 hubs
20 hubs
30 hubs
0 hubs
10 hubs
20 hubs
30 hubs
Retention of duplicates (%)
2 3 4 5 6 7 8
All
distances
Distance between enzymes
Supplementary figure 1. Influence of hub removal on the retention of duplicates in different metabolic networks. The results shown in the main text correspond to the analysis of EcoKegg reconstructed eliminating the top 20 hubs. Here we show the frequencies obtained for other metabolic networks (RefKegg, EcoCyc and MetaCyc) eliminating different number of hubs. The detection of retained duplicates and significance tests were repeated each time. In all plots histogram bars represent actual frequencies. Red circles represent the expected frequencies, +/- 3 , using 1000 null “Maslov-Sneppen” models
