# Supplementary material for: A network perspective on the evolution of metabolism by gene duplication
Source: Genome Biol. 2007 Feb 27;8(2):R26. doi: 10.1186/gb-2007-8-2-r26 (PMC1852415; doi:10.1186/gb-2007-8-2-r26)
Supplement: Additional data file 5 — Controls for the multidomain enzymes, the criteria of chemical similarity, and the method used to detect duplicates. [file gb-2007-8-2-r26-S5.ppt]

## Slide 1
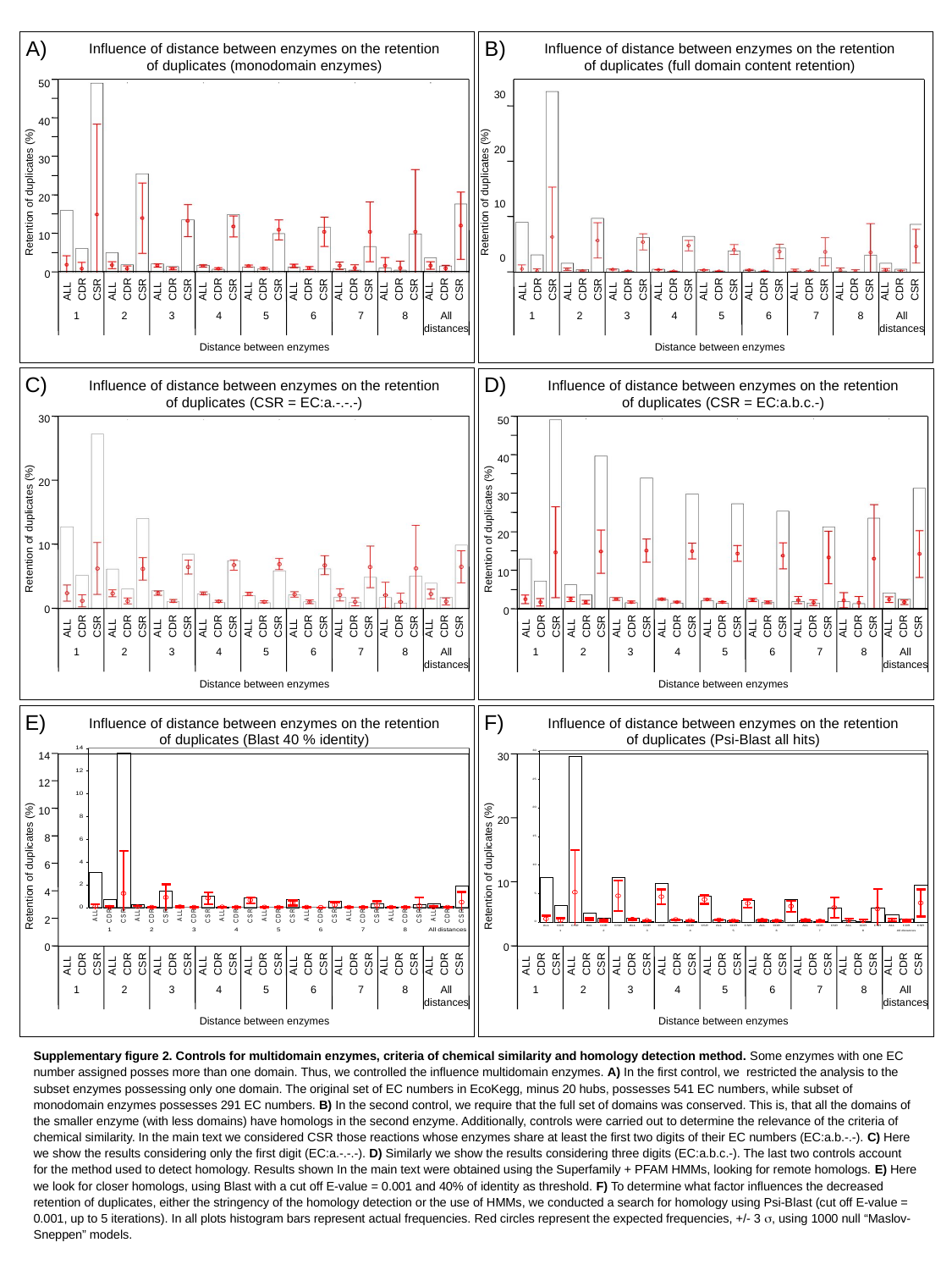

A)
B)
Influence of distance between enzymes on the retention
of duplicates (monodomain enzymes)
50
40
30
20
10
0
Retention of duplicates (%)
ALL
CDR
CSR
ALL
CDR
CSR
ALL
CDR
CSR
ALL
CDR
CSR
ALL
CDR
CSR
ALL
CDR
CSR
ALL
CDR
CSR
ALL
CDR
CSR
ALL
CDR
CSR
2 3 4 5 6 7 8
All
distances
Distance between enzymes
Influence of distance between enzymes on the retention
of duplicates (full domain content retention)
30
20
10
0
Retention of duplicates (%)
ALL
CDR
CSR
ALL
CDR
CSR
ALL
CDR
CSR
ALL
CDR
CSR
ALL
CDR
CSR
ALL
CDR
CSR
ALL
CDR
CSR
ALL
CDR
CSR
ALL
CDR
CSR
2 3 4 5 6 7 8
All
distances
Distance between enzymes
C)
Influence of distance between enzymes on the retention
of duplicates (CSR = EC:a.-.-.-)
30
20
10
0
Retention of duplicates (%)
ALL
CDR
CSR
ALL
CDR
CSR
ALL
CDR
CSR
ALL
CDR
CSR
ALL
CDR
CSR
ALL
CDR
CSR
ALL
CDR
CSR
ALL
CDR
CSR
ALL
CDR
CSR
2 3 4 5 6 7 8
All
distances
Distance between enzymes
D)
Influence of distance between enzymes on the retention
of duplicates (CSR = EC:a.b.c.-)
50
40
30
20
10
0
Retention of duplicates (%)
ALL
CDR
CSR
ALL
CDR
CSR
ALL
CDR
CSR
ALL
CDR
CSR
ALL
CDR
CSR
ALL
CDR
CSR
ALL
CDR
CSR
ALL
CDR
CSR
ALL
CDR
CSR
2 3 4 5 6 7 8
All
distances
Distance between enzymes
E)
F)
Influence of distance between enzymes on the retention
of duplicates (Psi-Blast all hits)
30
20
10
0
Retention of duplicates (%)
ALL
CDR
CSR
ALL
CDR
CSR
ALL
CDR
CSR
ALL
CDR
CSR
ALL
CDR
CSR
ALL
CDR
CSR
ALL
CDR
CSR
ALL
CDR
CSR
ALL
CDR
CSR
2 3 4 5 6 7 8
All
distances
Distance between enzymes
Influence of distance between enzymes on the retention
of duplicates (Blast 40 % identity)
14
12
10
8
6
4
2
0
Retention of duplicates (%)
ALL
CDR
CSR
ALL
CDR
CSR
ALL
CDR
CSR
ALL
CDR
CSR
ALL
CDR
CSR
ALL
CDR
CSR
ALL
CDR
CSR
ALL
CDR
CSR
ALL
CDR
CSR
2 3 4 5 6 7 8
All
distances
Distance between enzymes
Supplementary figure 2. Controls for multidomain enzymes, criteria of chemical similarity and homology detection method. Some enzymes with one EC number assigned posses more than one domain. Thus, we controlled the influence multidomain enzymes. A) In the first control, we restricted the analysis to the subset enzymes possessing only one domain. The original set of EC numbers in EcoKegg, minus 20 hubs, possesses 541 EC numbers, while subset of monodomain enzymes possesses 291 EC numbers. B) In the second control, we require that the full set of domains was conserved. This is, that all the domains of the smaller enzyme (with less domains) have homologs in the second enzyme. Additionally, controls were carried out to determine the relevance of the criteria of chemical similarity. In the main text we considered CSR those reactions whose enzymes share at least the first two digits of their EC numbers (EC:a.b.-.-). C) Here we show the results considering only the first digit (EC:a.-.-.-). D) Similarly we show the results considering three digits (EC:a.b.c.-). The last two controls account for the method used to detect homology. Results shown In the main text were obtained using the Superfamily + PFAM HMMs, looking for remote homologs. E) Here we look for closer homologs, using Blast with a cut off E-value = 0.001 and 40% of identity as threshold. F) To determine what factor influences the decreased retention of duplicates, either the stringency of the homology detection or the use of HMMs, we conducted a search for homology using Psi-Blast (cut off E-value = 0.001, up to 5 iterations). In all plots histogram bars represent actual frequencies. Red circles represent the expected frequencies, +/- 3 , using 1000 null “Maslov-Sneppen” models.
